# Effects of an Integrated Neurofeedback System with Dry Electrodes: EEG Acquisition and Cognition Assessment

**DOI:** 10.3390/s18103396

**Published:** 2018-10-11

**Authors:** Guangying Pei, Jinglong Wu, Duanduan Chen, Guoxin Guo, Shuozhen Liu, Mingxuan Hong, Tianyi Yan

**Affiliations:** 1 Key Laboratory of Convergence Medical Engineering System and Healthcare Technology, The Ministry of Industry and Information Technology, Beijing Institute of Technology, Beijing 100081, China; pei_guangying@bit.edu.cn (G.P.); wujl@bit.edu.cn (J.W.); 2 School of Life Science, Beijing Institute of Technology, Beijing 100081, China; duanduan@bit.edu.cn (D.C.); 3120181375@bit.edu.cn (G.G.); 3 Valley Christian High School, San Jose, CA 55101, USA; liushuozhen2002@gmail.com; 4 School of Life Science and Medicine, Dalian University of Technology, Liaoning 124221, China; xiaoyaozuiyuehmx@foxmail.com

**Keywords:** neurofeedback integrated system, dry electrode, alpha band training, working memory

## Abstract

Electroencephalogram (EEG) neurofeedback improves cognitive capacity and behaviors by regulating brain activity, which can lead to cognitive enhancement in healthy people and better rehabilitation in patients. The increased use of EEG neurofeedback highlights the urgent need to reduce the discomfort and preparation time and increase the stability and simplicity of the system’s operation. Based on brain-computer interface technology and a multithreading design, we describe a neurofeedback system with an integrated design that incorporates wearable, multichannel, dry electrode EEG acquisition equipment and cognitive function assessment. Then, we evaluated the effectiveness of the system in a single-blind control experiment in healthy people, who increased the alpha frequency band power in a neurofeedback protocol. We found that upregulation of the alpha power density improved working memory following short-term training (only five training sessions in a week), while the attention network regulation may be related to other frequency band activities, such as theta and beta. Our integrated system will be an effective neurofeedback training and cognitive function assessment system for personal and clinical use.

## 1. Introduction

Neurofeedback is regarded as a method to regulate one’s brain activity to directly alter relevant behaviors and neural mechanisms of cognition, which provides not only a method for cognitive enhancement in healthy subjects but also a significant therapeutic tool to normalize patient’s brain activity during rehabilitation [1]. Considering that the therapeutic potential for functional magnetic resonance imaging (fMRI) is severely limited by poor temporal resolution, as well as, the high cost, lack of portability and low reliability, neurofeedback based on the EEG is becoming widely adopted [2]. In view of the increased use of EEG neurofeedback, there is an urgency to reduce the discomfort and preparation time needed for participants to engage in neurofeedback training and a need for cognitive function to be evaluated easily and effectively after neurofeedback training [3]. An integrated wearable neurofeedback device can avoid the disadvantages of complex operations and training venues and has better practicability and potential for widespread personal and clinical use. Currently, hydrogel-based “wet” EEG electrodes are used as training electrodes adopted by most neurofeedback systems to provide adequate EEG signals. However, using these wet electrodes requires preparation of the skin and direct application of a conductive, water-based gel to obtain suitable EEG performance. In addition to the relatively long and laborious setup times, the gel may cause a large reduction in the signal-to-noise ratio. Shorts can also occur between neighboring recording sites due to sweat or due to smearing of the conductive gel. The EEG signal may degrade or even disappear as the gel dries due to the relatively high volatility of water at the temperatures experienced by the human scalp [4]. Aside from affecting the electrical properties, drying gel can cause discomfort from abrasion of the outer skin layer and/or allergic reactions [5]. For these reasons, wet electrodes are not an ideal candidate for long-term EEG neurofeedback consisting of many sessions [6]. Currently, dry and wireless EEG headsets allow subjects to move around without artifacts and allow participants to be cooperative while awake and without discomfort. Many of the problems noted above can be minimized by using dry electrode systems, and some recent studies have demonstrated working prototypes [7]. Advances in dry-electrode electroencephalography (EEG) and wireless integrated acquisition systems have spurred increased development of a new generation of wearable, mobile applications using EEG for real-world cognitive state monitoring, clinical diagnostics and therapeutics, and brain-computer interfaces (BCI), among others [8]. Considering the individual differences between subjects, optimal subsets of channels usually vary across subjects, which may still be needed for neurofeedback training or BCI applications. In addition, measuring interactions at the level of cortical sources, rather than sensors, can offer increased interpretability while reducing confounding factors of volume conduction [9]. However, few detailed reports on the associated neurofeedback systems are appropriate for a multichannel dry EEG electrode that covers the whole brain, although some dry electrode devices are currently used for neurofeedback, such as NeuroSky^®^, which only adopts frontal electrodes [10], and the Emotiv EPOC headset, which has 14 channels but does not provide certain electrodes on the central site of the brain [11]. Therefore, providing a neurofeedback system that is appropriate for multichannel electrodes will be more personalized and higher performance for clinical treatment and individual training.

Cognitive assessment is a valuable clinical skill for accurately estimating functional ability and evaluating the cognitive state after neurofeedback training [12]. A wide range of tools have been developed to perform cognitive assessments, which mainly include cognitive behavioral tasks, such as visual and verbal memory tests, backward digit span tasks and operation span tasks, and clinical scales, such as the Mini Mental State Examination (MMSE), Montreal Cognitive Assessment (MoCA) and Cambridge Cognitive Examination (CAMCOG), to demonstrate the efficacy and performance after neurofeedback training [13,14]. The appropriate choices depend on time available and the purpose of the assessment. Currently, some commercial software such as the Automated Neuropsychological Assessment Metrics (ANAM) [15] and CAMCI-Research [16], which provide computer-based tests or standardized neuropsychological tests modified for computer administration to assess cognitive change. E-prime for experimental design and behavioral data collection is widely used in clinical research [17]. Furthermore, some studies have also shown that cognitive training can improve cognition, such as memory and attention, and has a good synergistic effect on neurofeedback training [18]. However, there is no system that can provide cognitive function assessments and neurofeedback training at the same time; such a system would be able to evaluate the change of cognitive capacity in real time and provide a platform of task-based neurofeedback training.

Recently, people have suggested that abnormal alpha activity, which scalp EEG can collect in the frequency range 8–12 Hz, was associated with mental disorders [19], such as depression and anxiety. Alpha activity was suppressed with maturation and decreased after 40 years [20]. Therefore, alpha has been one of the most common protocols for neurofeedback training to improve personal cognitive performance [21]. Recent studies discovered that alpha activity of the frontoparietal region was also correlated to one’s intelligence [22] and memory performance [14]. The neurofeedback training (NFT) of the 8–12 Hz amplitude indicated that the alpha rhythm in the frontoparietal region enhanced both working memory [23] and episodic memory [14]. Another classic hypothesis was that the alpha rhythm was linked to cortical inhibition [24], which was especially effective at inhibiting unnecessary processes that conflicted with ongoing processing tasks. Thus, increased alpha activity may improve one’s attention by lowering the influence of distracting stimuli, which is also considered an effective training protocol for patients with attention deficit hyperactive disorder (ADHD) [25]. Because adjustment of alpha rhythm activity can improve both attention and memory at the same time by short-term neurofeedback training, it is worth exploring in healthy and elderly individuals with cognitive decline.

In this paper, we describe an integrated neurofeedback system that combines multichannel dry electrode EEG acquisition equipment and neurofeedback training software with cognitive function assessment. With this system, we used a single-blind control design to explore memory and attention performance after the regulation of alpha (8–12 Hz) power by short-term neurofeedback training. We found that the healthy subjects in the neurofeedback (NF) group had better performance in a working memory task compared with that in the sham-NF group, but there was no effect on episodic memory and alerting, orienting, and executive attention network tasks. Our integrated neurofeedback system was able to improve cognitive function and is expected to be used in clinical research and personal training.

## 2. Materials and Methods

### 2.1. Subjects

Twenty healthy volunteer subjects with normal or corrected-to-normal vision were recruited from the Beijing Institute of Technology to participate in the study, and participants were excluded for any history of medical (e.g., diabetes, cancer, etc.), neurologic (e.g., stroke, brain injury, etc.) or psychiatric conditions (depression, neurogenetic disorders, attention deficit hyperactivity disorder, etc.) that are known to affect cognition. The study was approved by the Beijing Institute of Technology Review Board. All subjects provided written informed consent. This included 10 subjects in each of the following groups: NF group: alpha frequency neurofeedback group; Sham-NF group: random 4 Hz neurofeedback group. Subjects in both groups received the same scripted instructions. All subjects had not participated in similar neurofeedback training before.

### 2.2. Neurofeedback System

**Figure 1:**
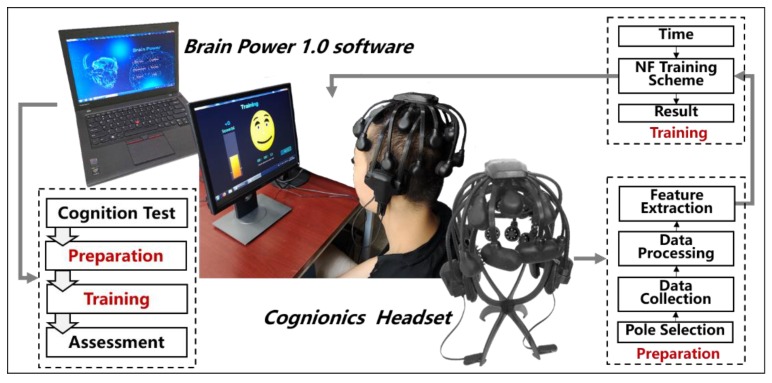
Neurofeedback system.

Our neurofeedback system primarily contained EEG collection equipment and a neurofeedback training system (Brain Power 1.0). During the experiment, participants were seated in a dimly lit room and used this system to complete the training (Figure 1). We adopted the latest mobile EEG technology (Quick-20, Cognionics, San Diego, CA, USA) [26], which is widely used in various EEG and BCI research protocols [27,28]. It was equipped with dry EEG sensors, which were specifically optimized for ease of use, and users could self-don the headset in approximately three minutes while still providing quality raw signals as well as time-averaged ERP responses that showed a high correlation (>0.9) between the wet and dry signals [29,30]. The Brain Power system was designed by us and consisted of a cognitive function test, a preparation phase, neurofeedback training and EEG data assessment functional modules. The system extracted the characteristic parameters of the main autonomous rhythm components in the EEG signal from delta to gamma rhythm and generates different types of visual feedback information to the subjects. The system was based on a brain-computer interface system framework, with multithread design, complete real-time processing of EEG signals and EEG feature extraction, feedback control, feedback information selection and feedback through a visual interface interaction design [31]. The software provided the memory and attention training protocols, performed the neurofeedback and used a custom module of training, which would allow the researcher to select a specific frequency or ratio to regulate, fifteen sets of visual feedback signal protocols were matched to personalized neurofeedback training. Furthermore, the cognitive function assessment was integrated and allowed the participant to complete the cognitive behavioral test for cognitive ability assessment, which provided randomized stimuli in tasks that are well-established cognitive measures, and it recorded the accuracy and the timing of the responses with millisecond sensitivity. This system will allow researchers to design personal protocols by selecting and arranging tasks to run from the battery of tests provided. Above all, this integrated system provided a neurofeedback training and effect evaluation platform.

### 2.3. Experimental Protocol

#### 2.3.1. Experimental Design

**Figure 2:**
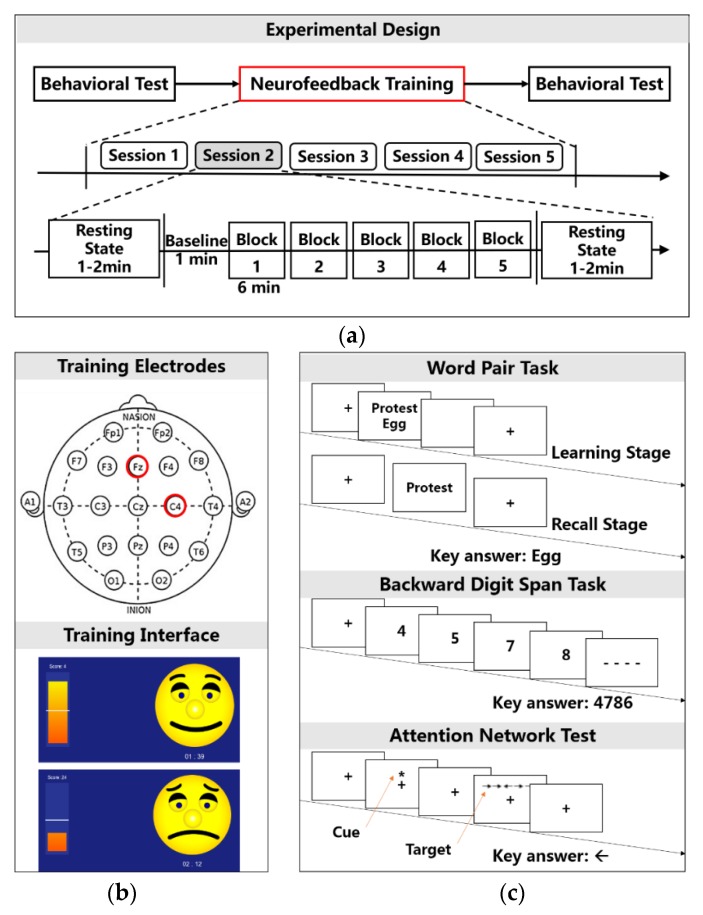
Flow diagram of the experiment for subjects. (**a**) Experimental design includes the behavioral test and neurofeedback training that covered five training sessions. There were six blocks that hold six minutes for each one in a session; (**b**) The neurofeedback protocol adopts Fz and C4 as training electrodes and the “face” dynamic picture as the training interface; (**c**) Behavioral tests include word pair task, backward digit span task and attention network test.

Subjects completed all of the behavioral tests and neurofeedback training. Behavioral tests were performed before and after neurofeedback training. Neurofeedback training included five sessions on different days. All subjects completed the study within 5–7 days. Each session contained a block of a 2 min EEG baseline recording followed by six training blocks of 6 min each with a 30 s break for rest [14,32]. Before and after each session of neurofeedback training, spontaneous resting EEG was recorded for 1–2 min with eyes opened (Figure 2a).

#### 2.3.2. Neurofeedback Protocol

Subjects in the NF group had alpha power as the feedback signal, and in the Sham-NF group, we chose a random 4 Hz band in the range of the 4–45 Hz frequency as the feedback signal. The Fz and C4 electrodes were used as training channels in two groups. We used the power of the target frequency band as the feedback feature during the neurofeedback training and then converted the visual signal as the chart and dynamic picture to the subjects. Subjects can learn to control the EEG activity in specific frequency bands based on quantitative EEG activities on a computer screen [33]. Here, picture “face” can be changed into “smiling” or “crying”; in particular, when the target frequency power was greater than the baseline, “smiling face” was awarded to subjects, otherwise “crying face”, appeared as shown in Figure 2b.

#### 2.3.3. Behavioral Tests

In our study, we assessed episodic memory, working memory, and attention network processes in the pretest and posttest periods relative to neurofeedback training. The word pair task assessed two episodic memory processes, that is, learning and recall. The 80 pairs of Chinese words were supplied by a previous study [34]. Subjects first completed the learning stage of the task and then completed the other two tasks. The interval between the learning and recall stages of the task was approximately 40 min. In this study, the attention network test was approximately 35 min, and 5 min was used to complete the backward digit span task. Each correct answer in the word pair task received one point, with a maximum score of 80. A backward digit span task tested the capacity of working memory storage. Thirty trials were performed in this task. Each trial contained four to eight digits, and each digit lasted for 1 s. Every trial started by a fixation (1 s). Subjects entered the digits in reverse order. The maximum score was 180 points, and every corrected digit was 1 point. The attention network test (ANT), which was a combination of the cued reaction time (RT) and the flanker tasks were used to test the efficiency of the three attentional networks that included alertness, orientation and execution control functions [35]. Efficiency of the three attentional networks was assessed by measuring how response times were influenced by alerting cues, spatial cues, and flankers. Four cue conditions (no cue, center cue, double cue and spatial cue) and three flanker types (neutral, congruent and incongruent) were adopted in the task. An arrow appeared above or below a fixation point and may or may not be accompanied by flankers. The ANT required participants to determine whether a central arrow pointed left or right. The formal experiment consisted of 312 trials. There were 78 trials for each interference state and 104 occurrences of each target. Among them, the number of times the target appeared above and below the center point in the entire experiment and the number of times the target were directed to the left and right were equal. Different experimental conditions were mixed and presented randomly. There were 24 exercises before the formal experiment. Each experiment indicated correct or incorrect results. The alertness, orientation and execution control functions of the attention network were calculated by the difference in response time and are calculated as follows: (1) Alerting attention: Average RT (no cue)—Average RT (double cue); (2) Orienting attention: Average RT(central cue)—Average RT(spatial cue); (3) Executive attention: Average RT(incongruent)—Average RT(congruent). The above tasks were designed using the Cognition Test module of the Brain Power 1.0 system (Figure 2c).

### 2.4. EEG Acquisition and EEG Data Analysis

The Quick-20 dry-wireless headset (Cognionics, San Diego, CA, USA) was used to acquire signals from the brain. The device was connected through a USB isolator to the computer. Based on the International 10–20 system, the EEG signals were acquired from 19 channels (Fp1, Fp2, F7, F3, Fz, F4, F8, T3, C3, Cz, C4, T4, T5, P3, Pz, P4, T6, O1 and O2) with two reference electrodes (A1 and A2) at a frequency rate of 500 Hz. Before and after NF training, the resting EEG was recorded under similar circumstances. During the resting state collection, the subject was required to watch the fixation “cross” on the screen and remain awake without tampering with the equipment [36,37]. During raw EEG signal preprocessing, which was subjected to 0.5-Hz high-pass and 45-Hz low-pass infinite impulse response (IIR) filters. Considering that vertical eye elevation had no direct influence on alpha activity [38], the Artifact Subspace Reconstruction (ASR) filter was applied to the EEG signals and was designed to detect and remove high-amplitude data components (for instance, artifacts stemming from eye blinks, muscle, and sensor motion) of high amplitude relative to some artifact-free reference data, while recovering EEG background activity that lies in the subspace spanned by the artifact components [9,39] (formula details shown in (1)). Then, purified EEG signal processing was done online by short-time Fourier transform [40]. The power of the alpha (8–12 Hz) data was calculated by fast Fourier transform (FFT) and was updated every second during the NF training, and the reward and punishment statistics were performed every five seconds [41]. If the power data exceeded the threshold three times or above in five seconds, a reward display was given. During the baseline of training acquisition, using the same interactive interface, the dynamic changes of the “face” were random to give the same stimulation environment to the subjects.

The EEG data were analyzed off line with EEGLAB, an open source MATLAB toolbox for electrophysiological signal processing. During raw EEG signals preprocessing, which were subjected to 0.5-Hz high-pass and 45-Hz low-pass finite impulse response (FIR) filters. Then, 10,000 ms epochs of resting state EEG data were used to determine absolute EEG activity in the theta (4–7 Hz), alpha (8–12 Hz), alpha1 (8–10 Hz), alpha2 (10–12 Hz), beta (13–30 Hz) and gamma (30–45 Hz) frequency bands. For the artifact rejection, independent component analysis (ICA) was applied to the EEG signals, and the components responsible for the eye movements and blinks were rejected. Then, we obtained the purified EEG signals of each group of experimental subjects. A new estimation of the time-frequency (TF) energy based on the wavelet transform of the artifact-free data was used, which provided a better compromise between time and frequency resolutions than short-term Fourier transforms [42,43]. The artifact-free data were convoluted by complex Morlet’s wavelets, which have a Gaussian shape both in the time domain and in the frequency domain around its central frequency. The time-frequency analysis, which is based on the wavelet transform (formula details shown in (2)), can synchronously provide the variation of the EEG signal in the time domain and frequency domain [44]. The EEG time-frequency characteristics were calculated, and visualization was performed using a time-frequency map and topographic map:

(1) $$S_{clean}{\;=\;V\;\times \;V}^{T}M\;\times \;{\left({\left(V^{T}M\right)}_{truncated}\right)}^{†\;}{\times \;V}^{T\;}\times \;S$$

(2) $$W_{f}\left(a,b\right)=\int_{R}\varphi_{a,b}^{*}\left(t\right)f\left(t\right)dt.$$

### 2.5. Statistical Analysis

Mean relative powers of different frequency bands in the 1st to 5th sessions and accuracies of the backward digital span task and word pair task were analyzed by paired samples *t*-test within group and independent sample *t*-test across group. The relative power of the frequency band was computed according to formula (3). The performance of the attention network test was evaluated by paired samples *t*-test within group and independent sample *t*-test across group. Furthermore, an analysis of variance (ANOVA) was conducted with Neurofeedback (NF group, Sham-NF group) as between-subjects factors and Cue type (no cue, central cue, double cue, spatial cue) and Flanker type (neutral, congruent, incongruent) as within-subject factors. ANOVA was conducted to explore changes in three attentional networks before and after training. Post hoc simple effect tests were performed based on any significant interaction effects involving the factors of Neurofeedback or Phase. Statistical analyses were calculated using SPSS 19 (SPSS, Chicago, IL, USA). Data are expressed as the mean ± standard error of the mean. A two-tailed significance level was set at *p* < 0.05. The methods of statistical analysis mentioned above have been verified by other experimental studies [45]:

(3) $$\;Relative\;power\;of\;frequency\;band\;ratio=\frac{Absolute\;Resting\;state\;Power\;after\;NF}{Absolute\;Resting\;state\;Power\;before\;NF}$$

## 3. Results

### 3.1. Subject Information

The mean age of the participants in the NF group and Sham-NF group was 22.7 years (SD = 1.952) and 21.2 years (SD = 1.720). ANOVA results (*p* = 0.124) revealed no statistically significant difference between the two groups in age. Moreover, the distribution of gender and education was the same. The participants’ demographic features are presented in Table 1.

### 3.2. Neurofeedback Performance

**Figure 3:**
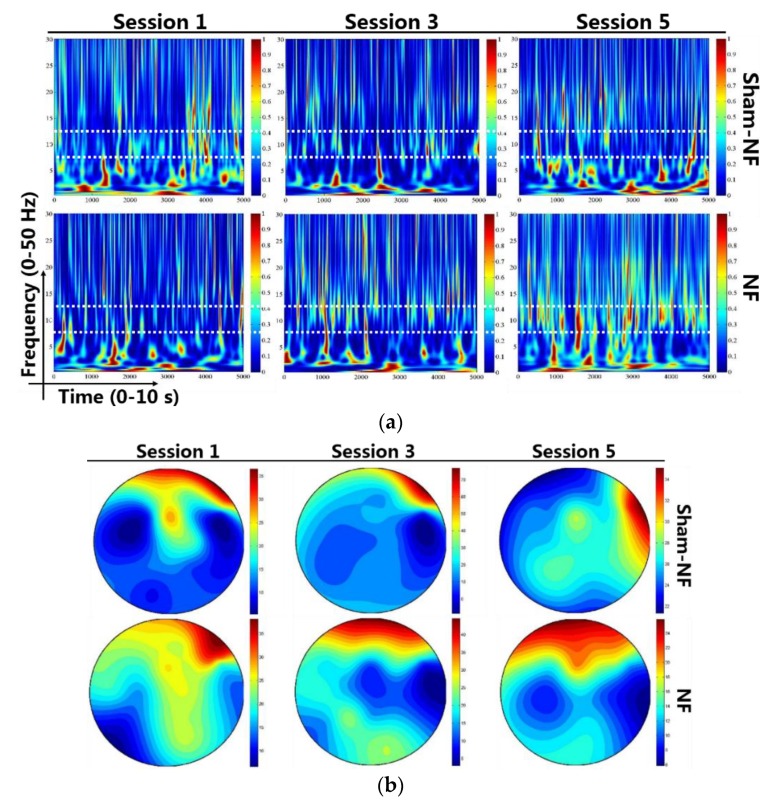
Time-frequency plots of EEG and averaged whole head EEG mapping of alpha frequency in the 1st, 3rd, and 5th sessions of a subject in the NF Group and Sham-NF group. (**a**) Time-frequency plots: The horizontal axis shows the time range, and the vertical axis shows the frequency range. The box with the dotted white line shows the induced power of the alpha (8–12 Hz) frequency band; (**b**) The distribution of the energy of alpha power in different sessions of a subject in the NF group and sham-NF group.

**Figure 4:**
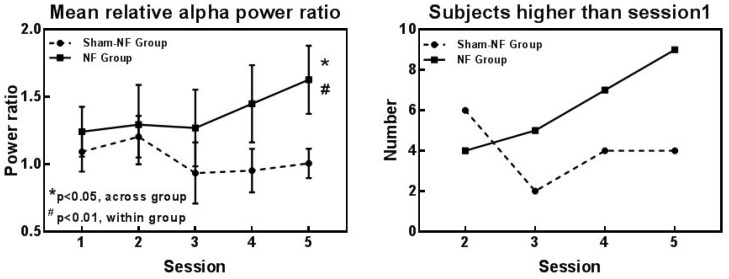
Changes in the relative alpha rhythm throughout neurofeedback training in the NF group and Sham-NF group. (**a**) Mean relative alpha power ratio in both groups. * represents (*p* < 0.05) when compared to the relative alpha power of session 5 of the sham-NF group. # represents (*p* < 0.01) when compared to the relative power of session 1 of NF group; (**b**) Numbers of subjects with relative alpha power higher than session 1 during training sessions 2–5 in both groups.

Several aspects of alpha rhythm showed progressive enhancement throughout the training sessions. The alpha rhythm time-frequency plots of a participant in the NF group and the Sham-NF group are shown in Figure 3a, and EEG traces of alpha and non-alpha recordings from both training and testing electrodes from a subject in NF group or Sham-NF group were showed in the Figure S1. The distribution of alpha power was mainly in the frontal area after neurofeedback training in the NF group, while the distribution in the sham-NF group was not obvious (Figure 3b). Subjects in the NF group were able to autonomically modulate the power of alpha rhythms by NF training. When the fifth session was compared to the first session, the relative power of alpha rhythm was higher (t = −2.905, *p* = 0.017) in the NF group, and not in the sham-NF group (t = 0.425, *p* = 0.681). As the number of training sessions increased, the relative power of the alpha rhythm (8–12 Hz) increased, and we found a significant difference across the groups in session 5 (t = −2.254, *p* = 0.043) (Figure 4a). Furthermore, in terms of individual outcomes, the number of subjects whose relative alpha power was higher than the first session gradually increased in the NF group along with the progress in the NF training, but in the sham-NF group, there was no rising trend (Figure 4b).

**Figure 5:**
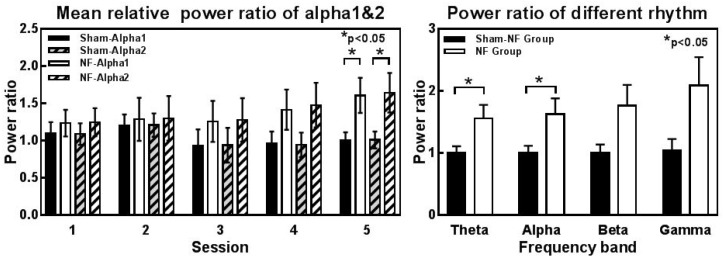
Changes in other rhythms throughout neurofeedback training in the NF and Sham-NF groups. (**a**) Relative power of Alpha1 (8–10 Hz) and Alpha2 (10–12 Hz) rhythms during training sessions in both groups. * represents (*p* < 0.05) across groups; (**b**) Relative power of different rhythms from theta to gamma in the 5th session. * represents (*p* < 0.05) across groups.

Meanwhile, we performed a more detailed analysis of alpha power and the other frequency band power. The lower alpha (alpha1: 8–10 Hz) and upper alpha (alpha2: 10–12 Hz) power were also significantly increased in the fifth session compared to those in the Sham-NF group (alpha1: t = −2.327, *p* = 0.037; alpha2: t = −2.198, *p* = 0.048) (Figure 5a). In addition to the alpha rhythm, we found that the relative mean theta rhythm power of the NF group showed enhancement in session 5 compared to the Sham-NF group (Figure 5b), and the details of the results of independent-sample *t*-tests for other rhythms’ relative power are shown in Table 2.

### 3.3. Cognitive Performance

#### 3.3.1. Memory Ability

**Figure 6:**
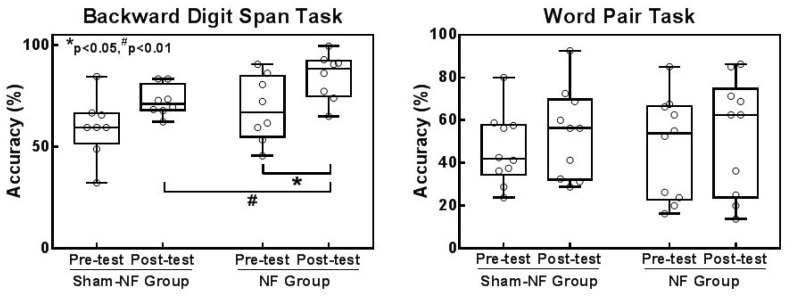
Changes in the accuracies of the backward digit span task and word pair task before and after training in two groups. (**a**) Accuracy in the backward digit span task of two groups; * represents (*p* < 0.05) across group and # represents (*p* < 0.05) within group; (**b**) Accuracy in the word pair task of two groups. The horizontal line in each box indicates the mean of the value of response time in each group. The hollow circles represent individual data.

To assess the changes in memory due to neurofeedback, response accuracy in the backward digit span task was measured to evaluate working memory and word paired task was used to evaluate episodic memory. Figure 6 shows the performance in the backward digit span task and word pair task separately in the two groups before and after NF training. The NF group showed extremely significant performance improvements in the backward digit span task after NF training (t = −4.283, *p* = 0.002) but did not show statistical significance in the Sham-NF group (t = −2.242, *p* = 0.052). Furthermore, the posttest accuracy of the NF group was significantly higher than that of the Sham-NF group (t = −2.201, *p* = 0.041), and there was no significant difference in accuracy between the two groups in the pretest (t = −0.994, *p* = 0.334) (Figure 6a). Additionally, the proportion of subjects in the Sham-NF group with reduced memory performance compared to the pretest was 20%, but no one in the NF group led to worse performance after NF training. In addition, we compared the accuracy of the word paired task within each group (NF Group: t = −1.714, *p* = 0.121; Sham-NF Group: t = −1.750, *p* = 0.114) and across groups (Pretest: t = −135, *p* = 0.894; Posttest: t = 0.081, *p* = 0.936), but no significant difference was found (Figure 6b). Table 3 shows the details of the mean accuracy of the two groups in the memory task before and after training.

#### 3.3.2. Attention Network

The mean accuracies (standard deviations) and reaction times (RTs) (standard deviations) for each of the cue-type and flanker-type conditions for the two groups in both pre- and post-training phases are presented in Table 4 and Table 5, respectively. For the accuracy data (Table 4), the overall accuracy on the ANT was over 99% in both groups, and an analysis of the overall number of errors showed that approximately 0.1% of all errors were wrong responses and omissions. For the RTs data (Table 5), the results of a 2 (Neurofeedback: NF group, Sham-NF group) × 2 (Phase: pretest, posttest) × (Cue type: no cue, central cue, double cue, spatial cue) × (Flanker type: neutral, congruent, incongruent) ANOVA were as follows. The main effects of Cue type (F(3, 76) = 8.982, *p* = 0.000) and Flanker type (F(2, 57) = 44.176, *p* = 0.000) were all significant. Specifically, the main effects of Cue type (F(3, 60) = 3.162, *p* = 0.025; F(3, 60) = 7.374, *p* = 0.000) and Flanker type (F(2, 27) = 14.479, *p* = 0.000; F(2, 27) = 38.642, *p* = 0.000) in the NF group and Sham-NF group were all significant. In view of the alertness, orientation and execution control functions of the attention network calculated by the difference in response time. We have analyzed in detail three attention network-related RTs data. All participants’ response times increased from the spatial cue, double cue, central cue, and no cue conditions, and the simple effects were also significant (no cue vs. double cue (*p* = 0.000), central cue vs. special cue (*p* = 0.050)). The RTs increased from the neutral flanker, congruent flanker, and incongruent flanker conditions, and the simple effects were significant (congruent flanker vs. incongruent flanker (*p* = 0.000)).

**Figure 7:**
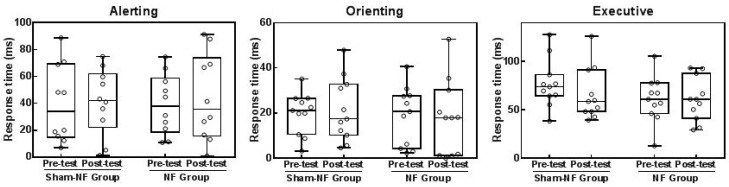
The response times (ms) scores of alerting, orienting and conflict in the pretest and posttest in both groups (**a**–**c**). The horizontal axis shows the group (NF and Sham-NF groups) and phase (pretest and posttest). The vertical axis shows the value of response times (ms). The horizontal line in each box indicates the mean of the value of response time in each group. The hollow circles represent personal data.

Figure 7 shows the efficiency of the three attentional networks in the Sham-NF group and NF group. In the NF group, compared to the pretest, although the neurofeedback training, three networks showed no significant variation (alertness: t = −0.616, *p* = 0.553; orientation: t = 0.147, *p* = 0.886; execution: t = −0.006, *p* = 0.995). In the Sham-NF group, the performance did not significantly improve (alertness: t = −0.131, *p* = 0.899; orientation: t = −0.229, *p* = 0.824; execution: t = 2.153, *p* = 0.06).

Based on these findings, NF training with alpha power enhancement of 5 sessions may improve working memory, while the performance of episodic memory and attention networks were not improved.

## 4. Discussion and Conclusions

Here we evaluated an integrated neurofeedback system, which was equipped with a multichannel dry electrode EEG collection system, by a classic neurofeedback training protocol that regulated the alpha power. The results showed that our system was able to successfully allow participants to perform neurofeedback training and cognitive performance testing. At the same time, we found that the successful upregulation of the alpha power effectively improved working memory, which is in line with results obtained by a previous study [34].

### 4.1. Integrated Neurofeedback System: Varying Interface, High Efficiency, Complete Functions and Wide Suitablity

Our system was able to cover all ranges of the frequency band from delta to gamma as protocols to be adjusted, and there are three types of interactive interfaces, including 15 kinds of visual schemes in detail. For the conversion of EEG signals to visual stimuli, which was used to provide effective EEG activity information for the subjects, including picture displays, 3D games and a 2D drawing game. Furthermore, each interface was equipped with a quantized histogram and a specific score display, which can more visually display the current EEG signal to subjects and allow them to better sense their EEG signals in turn. In this experiment, we chose the change of the “face” picture to show the dynamic of EEG signal or performance of the subject in the neurofeedback training. When the alpha power exceeded the threshold, the smiling face reflected that the positive emotion will be shown to reward the subject and add to their score, which can activate the reward mechanism in neurofeedback training [46]. Otherwise, the crying face is shown to punish the subject and to prompt them to change the regulation strategy. This is important for the motivation to the subject to best promote learning, not just to observe a biosignal correlate over a long period of time [47]. The adoption of 3D games and the current use of virtual reality games trying to provide more interactions with the virtual world through the BCI to receive the activity of the brain [48].

During this neurofeedback training, the use of dry electrodes did not bring any discomfort to the subjects during the entire process, and they completed an entire session of neurofeedback training, including device connection and wear, within one hour, which greatly increased the efficiency of training and achieved good training results in just five training sessions. At the same time, the cognitive function assessment within the experimental design provided by the system ensured the smooth transition to and from cognitive function testing. The functionality provided by this system is good for the combination of cognitive training and neurofeedback in the future [49], and some research using neurofeedback and testing in a cognitive task has achieved good performance in executive abilities [18].

### 4.2. Effects of System: Improve Working Memory by Intensifying Alpha Activity

In this study, we selected Fz and C4 electrodes as training electrodes. The C4 electrode was used to improve attention and memory in related neurofeedback training [14,50]. In frontal sites (Fz electrode), alpha activity might be caused by thalamic and anterior cingulate cortex activity, which addresses attention and working memory processing. Regulating the alpha amplitude in the central lobe can improve memory and that has been demonstrated [14]. Currently, the majority of studies often adopt several channels as training sites. Additionally, maintaining a minimal number of channels is essential for designing a portable brain–computer interface system for daily usage [51]. Additionally, reducing EEG channels and finding established locations in the head for electrode implementation can improve the performance and reduce the complexity of different BCI applications [52]. A few channel EEG signals were selected to be expanded into multichannel signals in the BCI system, which is viable, and the performance of EEG signals are stable over subjects and robust to artifacts [53,54]. Recently, single-channel BCI was successfully used in binary classification or multiclassification, especially for mental arithmetic versus letter imagination tasks [55]. Our system is able to support one-to-multi electrodes that cover the whole brain, including the frontal area, left and right temporal area, central area and occipital area in neurofeedback training and satisfy the need for an individualized protocol for optimum efficiency and portability.

Furthermore, we used the same process for behavioral experiments in two groups. Since episodic memory tasks need to time between tests, we performed the other two tasks after the learning stage of the task and finally performed the recall stage test. In the word-pair task, we used two Chinese vocabulary to test the performance separately before and after training, and the difficulty of test is consistent. To evaluate neurofeedback performance, the relative alpha power was analyzed because of the difference in baseline in each subject. The relative alpha power was higher in the NF group than in the Sham-NF group though five training sessions. The distribution of the alpha power was mainly concentrated in the frontal area in the NF group, which is related to the processing of working memory that neurophysiological studies have revealed [56]. Our behavioral results illustrated that working memory achieved better performance in the NF group.

However, there was no significant performance improvement in episodic memory, which may be due to the short training session just 5 days. Although the earlier studies have been based on 4 or 5 NFB sessions [57], studies in this research area are often based on approximately ten training sessions, and the most compelling effects of NFB on cognitive performance were observed when participants were subjected to an even greater number of training sessions [58]. Although the 12 sessions of neurofeedback training by increasing alpha power can effectively improve the episodic memory [14]. In addition, we also detected the activity of other frequency bands, especially for theta, beta and gamma, which are always the main parameters of the neurofeedback protocol, and the relative power of theta (4–8 Hz) as an adjacent frequency band of alpha improved after neurofeedback training, which was enhanced in the frontal region related to memory encoding and retrieval [59]. Considering that alpha1 is related to attention and our results have been upregulated, some studies have indicated that it inhibits the high theta band and improves the beta band to improve attention, particularly in ADHD. An alpha and theta intensive as a valid alternative for older populations to NF methodologies [50]. Therefore, improving the attention network may require the adjustment of activities in other frequency bands, such as theta and beta. From the perspective of the behavior test of the attention network, the alerting and orienting systems have been associated with the parietal and frontal lobes, and the executive control of attention activates midline frontal areas (anterior cingulate) and the lateral prefrontal cortex. Considering our training area of mainly focus on the frontal, eye movements might influence the performance of the test, although the work of Corbetta and associates has shown that overt and covert shifts of attention use the same anatomy [60]. It is not excluded that using other types of attention tests will be different. Here, there is no significant improvement in the performance of the attention network test.

### 4.3. Limitations and Further Research Direction

Of course, our system is constantly being updated and improved. Considering the cognitive load of the participants, we will add other sensory information presentation methods to meet the adjustment of various parameters at the same time in future work because the integration of sensory information will be more effective as information procession [61]. Some studies revealed that combined visual and auditory feedback modality achieved better performance than standard visual feedback in self-regulation neurofeedback or BCI performance, which may enable participants to communicate using their brain or focus the attention on the task and no longer be able to notice body movements and other disturbing activities [31,62,63]. Furthermore, haptic feedback did not present any artifacts to the classified brain signals and frees visual attention to other tasks, which will be required for BCI-application [64].

Neurofeedback has been discovered as a promising noninvasive tool for cognitive improvement and rehabilitation. In this study, adjusting alpha activity with neurofeedback training can improve memory for healthy subjects. Some strategies have been adopted during training. Recalling textual information and some positively impressive things, such as an unusually intense game or a happy thing. Some studies have mentioned that motor imagery as an assistant during neurofeedback training can regulate beta frequency band activity and improve motor symptoms in patients with Parkinson’s disease [65]. The specific regulation strategy that is effective for targeting frequency band regulation is worth investigating and as the supportive feedback signal in neurofeedback training improves in effectiveness and success rate of neurofeedback training in future work. The BCI-driven neurorehabilitation approach is an effective therapy to achieve functional recovery from motor-related disabilities, such as stroke [66]. Additionally, steady-state visually evoked potential EEG signals can be utilized to control a wheelchair for the disabled [67]. Neurofeedback systems based on BCI technology have the potential for neurorehabilitation and make BCI a promising tool for next-generation human-computer interaction (HCI) [68]. In addition to reducing suffering and improving quality of life, our system has the potential to advance our knowledge about the mechanisms of the nervous system [69]. For example, training target brain signals to regulate specific cognitive abilities and to clarify the neural mechanisms in clinical research.

## Figures and Tables

**Table 1:** Demographic characteristics of the participants.

Characteristics	NF Group (n = 10)	Sham-NF Group (n = 10)	*p* Value
Age (Mean ± SD ^1^), years	22.7 ± 1.952	21.2 ± 1.720	0.124
Gender (Female:Male)	(3:7)	(3:7)	-
Educational Level	BD ^2^:10	BD ^2^:10	-

^1^ SD: standard deviation; ^2^ BD: bachelor’s degree.

**Table 2:** Across group analysis of relative power in different rhythms.

Rhythm	Theta	Alpha	Beta	Gamma
Session 1	t(18) = −0.394, *p* = 0.698	t(18) = −0.626, *p* = 0.539	t(18) = −0.636, *p* = 0.533	t(18) = −0.580, *p* = 0.569
Session 2	t(18) = −0.244, *p* = 0.810	t(18) = −0.267, *p* = 0.793	t(18) = −0.257, *p* = 0.800	t(18) = −0.430, *p* = 0.672
Session 3	t(18) = −0.968, *p* = 0.346	t(18) = −0.920, *p* = 0.370	t(18) = −0.913, *p* = 0.373	t(18) = −0.969, *p* = 0.346
Session 4	t(18) = −1.384, *p* = 0.183	t(18) = −1.508, *p* = 0.149	t(18) = −1.436, *p* = 0.168	t(18) = −1.315, *p* = 0.205
Session 5	**t(12.668) = −2.355, *p* = 0.035**	**t(12.270) = −2.254, *p* = 0.043**	t(11.671) = −2.129, *p* = 0.055	t(11.998) = −2.136, *p* = 0.054

**Table 3:** Mean accuracies (standard deviations) according to memory task for each group.

Group	NF (Accuracy %)		Sham-NF (Accuracy %)	
Task name	pretest	posttest	pretest	posttest
BDST ^1^	68.45 ± 14.74	82.83 ± 10.87	61.5 ± 16.46	73.28 ± 8.39
WPT ^2^	47.5024.07	53.13 ± 27.05	46.25 ± 16.83	54.00 ± 20.73

^1^ BDST: backward digit span task; ^2^ WPT: word pair task.

**Table 4:** Mean accuracies (standard deviations) according to cue and flanker types for each group.

Group			Sham			NF	
Cue Type	Phase	Neutral	Congruent	Incongruent	Neutral	Congruent	Incongruent
No	Pre	549.5610	550.0533	647.6490	577.3071	597.9826	667.8678
		(70.8546)	(70.5773)	(72.3438)	(65.2450)	(97.4248)	(65.6699)
	Post	521.6632	531.1997	613.8091	566.6616	566.5036	649.5054
		(55.9449)	(57.9034)	(60.4480)	(91.2824)	(69.0719)	(87.6201)
Center	Pre	511.7615	546.6616	613.1631	566.7919	575.8866	631.1587
		(54.5679)	(72.1105)	(67.2868)	(77.0855)	(79.7234)	(79.3718)
	Post	507.9310	503.0821	558.6546	551.2491	536.5269	601.8069
		(41.4997)	(50.9089)	(58.4397)	(105.4994)	(83.6195)	(93.6850)
Double	Pre	512.0490	520.3047	595.0804	545.6622	557.6873	622.1264
		(47.4418)	(57.6369)	(54.8506)	(86.3291)	(75.0534)	(87.2844)
	Post	496.5916	493.1745	553.2295	520.5884	538.9169	590.0346
		(56.5859)	(58.8005)	(44.1290)	(85.6889)	(89.7205)	(98.3674)
Spatial	Pre	519.3953	511.8689	580.6556	534.2236	564.5059	618.8624
		(59.6872)	(54.3823)	(59.9480)	(57.9978)	(75.8159)	(101.8659)
	Post	479.0783	480.0095	545.9844	530.6068	530.2467	575.0067
		(48.3839)	(49.2976)	(61.1225)	(89.4958)	(78.6126)	(103.1026)

**Table 5:** Mean RTs (standard deviations) according to cue and flanker types for each group.

Group			Sham			NF	
Cue Type	Phase	Neutral	Congruent	Incongruent	Neutral	Congruent	Incongruent
No	Pre	0.9997	1.0000	0.9984	0.9994	0.9997	0.9984
		(0.0010)	(0.0000)	(0.0026)	(0.0013)	(0.0010)	(0.0022)
	Post	0.9987	1.0000	0.9994	1.0000	0.9994	0.9984
		(0.0016)	(0.0000)	(0.0013)	(0.0000)	(0.0013)	(0.0026)
Center	Pre	0.9997	1.0000	0.9984	0.9997	1.0000	0.9994
		(0.0010)	(0.0000)	(0.0026)	(0.0010)	(0.0000)	(0.0019)
	Post	0.9987	1.0000	0.9994	1.0000	1.0000	0.9990
		(0.0016)	(0.0000)	(0.0013)	(0.0000)	(0.0000)	(0.0021)
Double	Pre	0.9984	0.9997	0.9984	0.9997	1.0000	0.9981
		(0.0016)	(0.0010)	(0.0022)	(0.0010)	(0.0000)	(0.0026)
	Post	0.9981	1.0000	0.9981	0.9994	0.9997	0.9984
		(0.0026)	(0.0000)	(0.0021)	(0.0013)	(0.0010)	(0.0026)
Spatial	Pre	0.9984	0.9987	0.9974	0.9994	0.9990	0.9994
		(0.0030)	(0.0021)	(0.0024)	(0.0019)	(0.0021)	(0.0019)
	Post	0.9994	0.9997	0.9981	0.9984	0.9994	0.9981
		(0.0013)	(0.0010)	(0.0021)	(0.0030)	(0.0013)	(0.0026)

## Supplementary Materials

The following are available online at http://www.mdpi.com/1424-8220/18/10/3396/s1, Figure S1: EEG traces of alpha and non-alpha recordings from all electrodes of a subject in the NF group and Sham-NF group.
