# Supplementary material for: Effects of an Integrated Neurofeedback System with Dry Electrodes: EEG Acquisition and Cognition Assessment
Source: Sensors (Basel). 2018 Oct 11;18(10):3396. doi: 10.3390/s18103396 (PMC6211015; doi:10.3390/s18103396)
Supplement: Supplementary file 1 [file sensors-18-03396-s001.zip › sensors-357577-SI.pptx]

## Slide 1
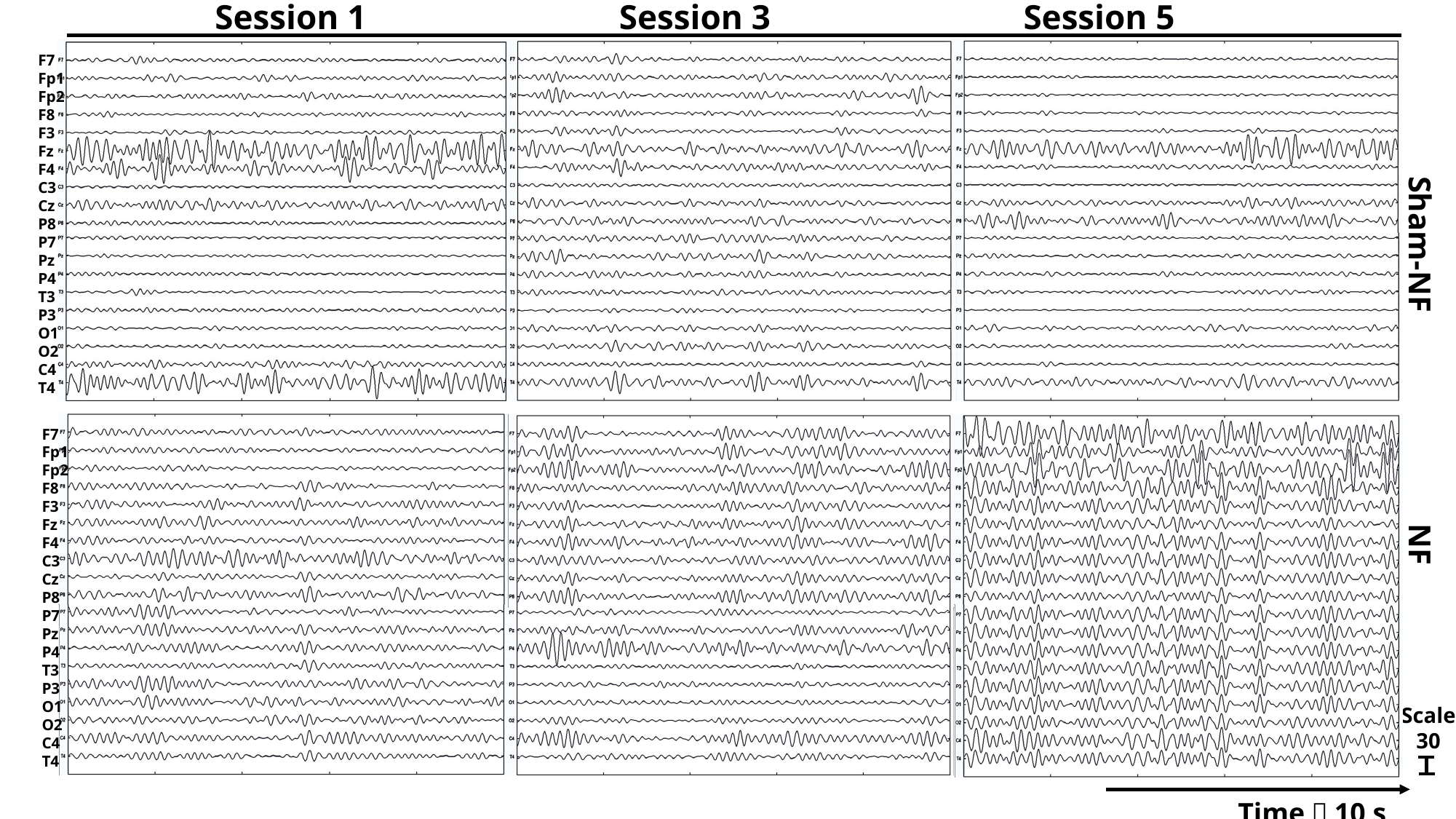

Session 1 Session 3 Session 5
F7
Fp1
Fp2
F8
F3
Fz
F4
C3
Cz
P8
P7
Pz
P4
T3
P3
O1
O2
C4
T4
F7
Fp1
Fp2
F8
F3
Fz
F4
C3
Cz
P8
P7
Pz
P4
T3
P3
O1
O2
C4
T4
Time：10 s
Sham-NF NF
Scale
30
